# Nationwide TREC/KREC-based newborn screening and clinical outcomes in Japan

**DOI:** 10.70962/jhi.20260083

**Published:** 2026-08-17

**Authors:** Tsubasa Nishinosono, Hideki Muramatsu, Manabu Wakamatsu, Nobuyuki Ishige, Junji Hanai, Takahiro Imaizumi, Masahiro Ueki, Yoji Sasahara, Toshinao Kawai, Yoji Uejima, Takahiro Yasumi, Satoshi Okada, Mariko Eguchi, Masataka Ishimura, Tomoyuki Mizukami, Masafumi Yamada, Hidenori Ohnishi, Go Tajima, Kohsuke Imai, Newborn Screening Working Group, Japanese Society for Immunodeficiency and Autoinflammatory Diseases, Hideki Muramatsu, Manabu Wakamatsu, Masahiro Ueki, Yoji Sasahara, Toshinao Kawai, Yoji Uejima, Takahiro Yasumi, Satoshi Okada, Mariko Eguchi, Masataka Ishimura, Tomoyuki Mizukami, Masafumi Yamada, Hidenori Ohnishi, Kohsuke Imai

**Affiliations:** 1 Department of Pediatrics, Nagoya University Graduate School of Medicine, Nagoya, Japan; 2 Department of Pediatric Critical Care, Shizuoka Children’s Hospital, Shizuoka, Japan; 3 Newborn Screening Working Group, Japanese Society for Immunodeficiency and Autoinflammatory Diseases, Tokyo, Japan; 4 Division of Newborn Screening, Tokyo Health Service Association, Tokyo, Japan; 5 Hokkaido Pharmaceutical Association Public Health Examination Center, Sapporo, Japan; 6 Department of Clinical Research Education, Nagoya University Graduate School of Medicine, Nagoya, Japan; 7 Department of Pediatrics, Hokkaido University Graduate School of Medicine, Sapporo, Japan; 8 Department of Pediatrics, Tohoku University Graduate School of Medicine, Sendai, Japan; 9 Department of Immunology, National Center for Child Health and Development, Tokyo, Japan; 10 Division of Infectious Diseases and Immunology, Saitama Prefectural Children’s Medical Center, Saitama, Japan; 11 Japan Environment and Children’s Study Kyoto Regional Center, Kyoto University Graduate School of Medicine, Kyoto, Japan; 12 Department of Pediatrics, Hiroshima University Graduate School of Biomedical and Health Sciences, Hiroshima, Japan; 13 Department of Pediatrics, Ehime University Graduate School of Medicine, Matsuyama, Japan; 14 Department of Pediatrics, Graduate School of Medical Sciences, Kyushu University, Fukuoka, Japan; 15 Department of Pediatrics, National Hospital Organization Kumamoto Medical Center, Kumamoto, Japan; 16 Department of Food and Human Wellness, Rakuno Gakuen University, Ebetsu, Japan; 17 Department of Pediatrics, Gifu University Graduate School of Medicine, Gifu, Japan; 18 Division of Neonatal Screening, Research Institute, National Center for Child Health and Development, Tokyo, Japan; 19 Department of Pediatrics, National Defense Medical College, Tokorozawa, Japan

## Abstract

Large-scale real-world data on T-cell receptor excision circle (TREC)– and κ-deleting recombination excision circle (KREC)–based newborn screening for inborn errors of immunity remain limited. In this nationwide cohort, we evaluated Japan’s program (2020–2025), including 1,380,634 newborns. Of 869 referred infants (0.06%), 40 were diagnosed, including 15 with severe combined immunodeficiency (SCID), 16 with X-linked agammaglobulinemia or other agammaglobulinemias, and nine with severe T cell lymphopenia. After a median follow-up of 18 mo, overall survival was 98%. All infants with SCID survived; 14 underwent hematopoietic cell transplantation with sustained engraftment. Referral rates were 0.04% for TREC and 0.03% for KREC. Repeat sampling was associated with lower referral rates (0.05 vs. 0.56%). Referral rates varied across assay kits and laboratory practices, reflecting interlaboratory heterogeneity in a decentralized screening system. The transition from self-paid to public funding increased coverage from 77.1 to 97.7%. These findings support TREC/KREC-based screening and highlight the need for standardized algorithms and sustained public funding for equitable nationwide implementation.

## Teaser

Nishinosono et al. show that nationwide TREC/KREC newborn screening in Japan demonstrates that large-scale programs can achieve favorable outcomes while revealing practical implementation challenges. Public funding expands coverage, whereas interlaboratory variability highlights the need for standardized cutoffs and repeat-sampling algorithms for equitable nationwide implementation.

## Graphical Abstract

**Figure:**
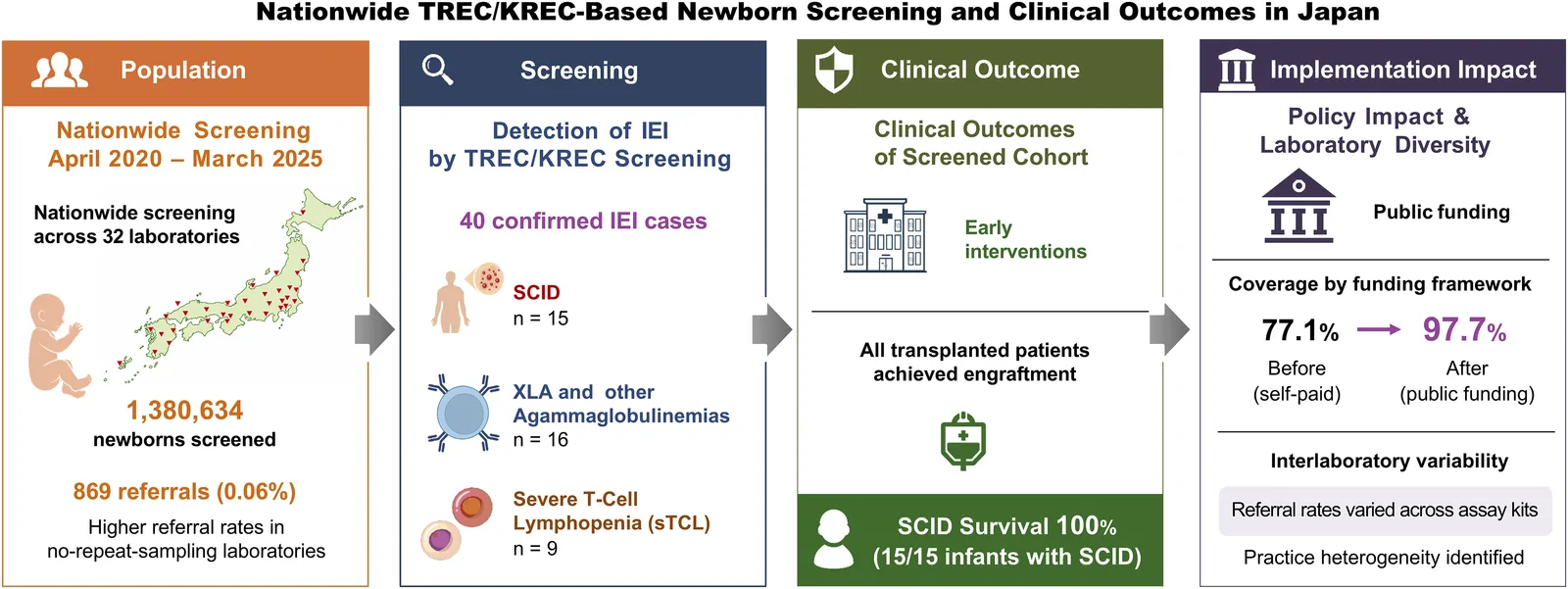

## Introduction

Newborn screening (NBS) to identify congenital disorders before symptom onset is an essential public health initiative that enables timely treatment and reduces morbidity and mortality (1, 2). The development of molecular assays for T cell receptor excision circles (TRECs) and κ-deleting recombination excision circles (KRECs) has made DNA-based NBS for inborn errors of immunity (IEIs) possible (3, 4, 5, 6, 7). TREC/KREC-based NBS primarily targets severe combined immunodeficiency (SCID) and X-linked agammaglobulinemia (XLA), while severe T cell lymphopenia (sTCL) may also be detected as a secondary finding (3, 4). SCID, the most severe IEI, causes fatal infections in the first year of life if untreated (8, 9). Hematopoietic cell transplantation (HCT) can markedly improve survival, especially when performed before 3.5 mo of age (10, 11, 12, 13). Infants with XLA are susceptible to recurrent bacterial infections when maternal antibodies decline at around 4–6 mo of age. Early diagnosis allows timely initiation of immunoglobulin replacement therapy (IGRT), helping to prevent infections and support normal development (14, 15).

Although TREC-based NBS is now widely implemented, adoption of TREC/KREC-based NBS has been slower due to concerns about false-positive rates, cost-effectiveness, and limited evidence on long-term outcomes (16, 17, 18). Nevertheless, nationwide or regional TREC/KREC-based NBS programs have now been implemented in countries across Europe and other regions (1, 16, 18, 19, 20, 21, 22, 23, 24, 25, 26, 27, 28, 29). In Japan, a TREC-based NBS pilot study began in Aichi Prefecture in 2017. This was followed by the introduction of an optional self-paid TREC/KREC-based NBS program in 2020, which has since expanded nationwide (17, 30, 31). This program has been implemented in a decentralized manner at the prefectural and municipal levels, with regional public laboratories independently selecting assay platforms, defining cutoff values, and setting prices. As a result, testing procedures differ across regions. In 2024, the Japanese government’s Children and Families Agency launched a national pilot project to provide publicly funded NBS for SCID, which was introduced sequentially across participating regions. As of March 2025, 37 of 67 administrative regions in Japan had joined this national pilot project.

In this nationwide cohort study, we analyzed data from more than 1.3 million newborns who underwent TREC/KREC-based screening in Japan. Our aims were to evaluate the implementation status and clinical impact of TREC/KREC-based NBS, as well as to assess interassay and interlaboratory variability in screening performance within a nationally unified population. These findings may help inform efforts to implement and standardize TREC/KREC-based NBS.

## Results

### Screening outcomes

**Figure 1.**
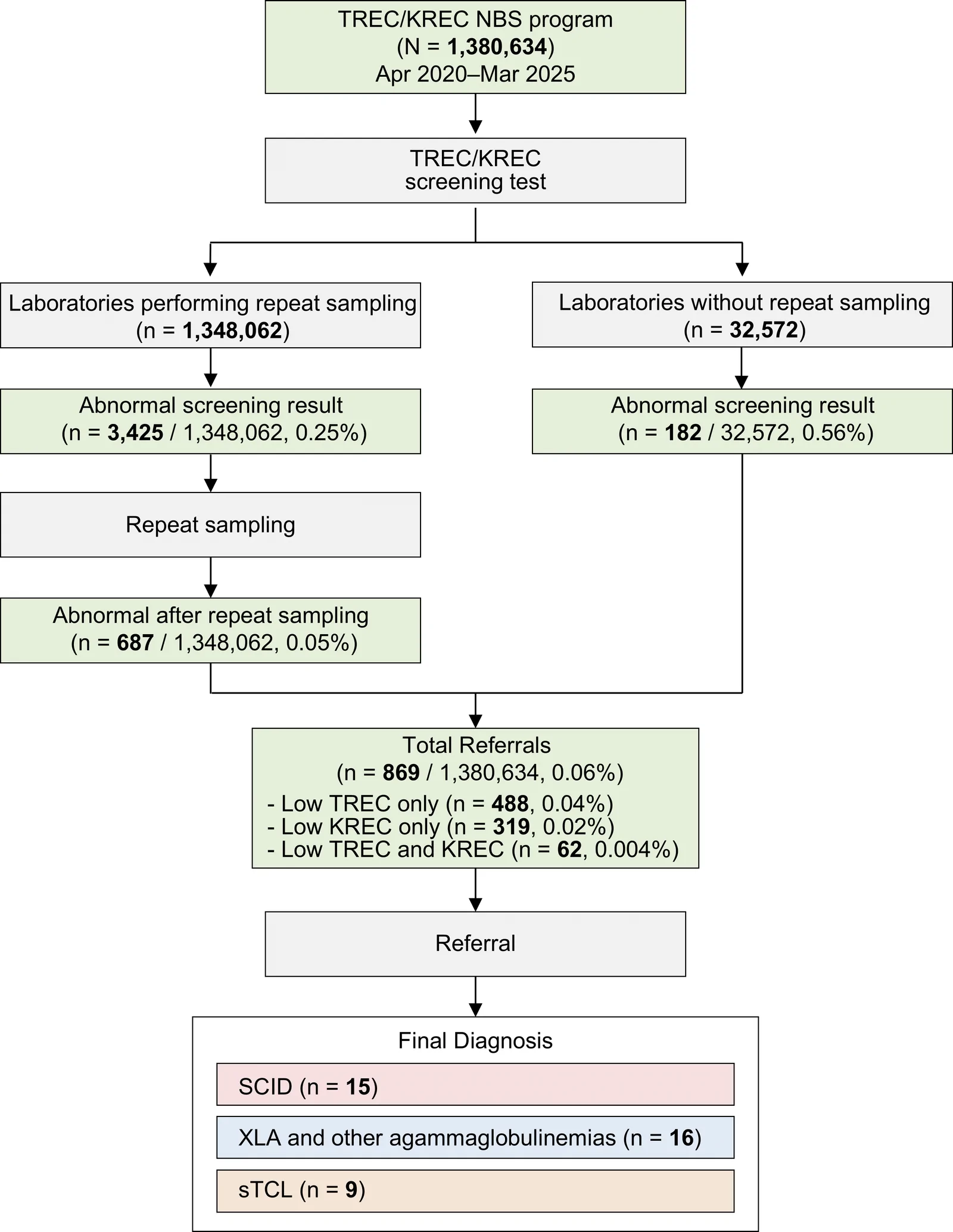
**Flow diagram of NBS for** **IEIs** **in Japan (April 2020–March 2025).** The diagram shows the total number of newborns screened, those requiring repeat sampling, referrals to IEI centers, and the final confirmed diagnoses. 30 laboratories performed repeat sampling. Two laboratories directly referred infants with low initial TREC/KREC values.

**Figure S1.**
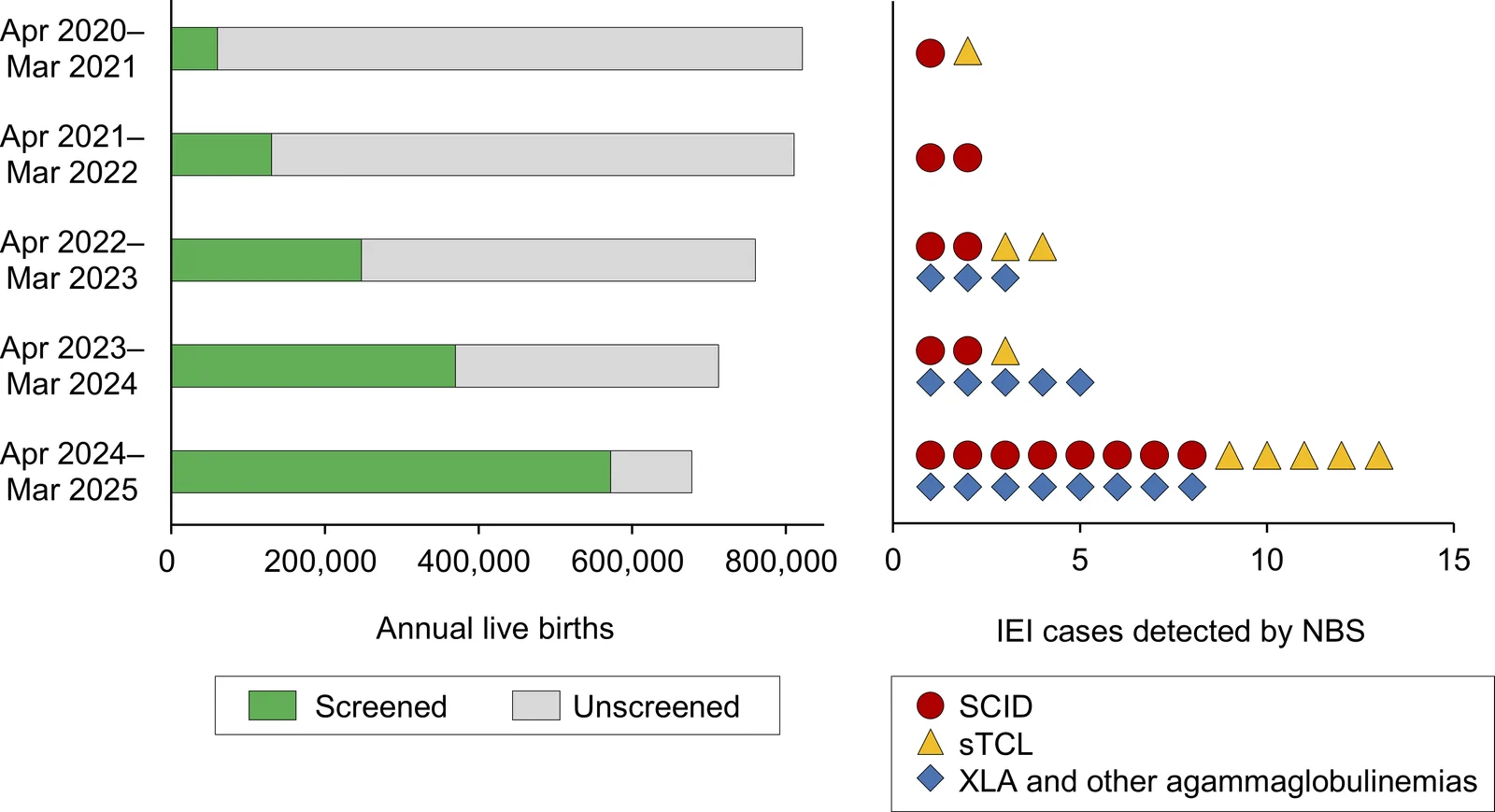
**Annual population-based coverage of TREC/KREC-based NBS in Japan (April 2020–March 2025).** The stacked bars represent annual live births, divided into infants screened with TREC/KREC and unscreened infants. Symbols on the right indicate the number of IEI cases detected by NBS each year. SCID, severe combined immunodeficiency; sTCL, severe T cell lymphopenia; XLA, X-linked agammaglobulinemia.

A total of 1,380,634 newborns underwent TREC/KREC screening between April 2020 and March 2025 (Fig. 1). During this period, the geographic coverage of the NBS program progressively expanded across Japan. In the final fiscal year (April 2024–March 2025), 572,175 of 677,922 live births were screened, corresponding to a population-based coverage of 84.4% (Fig. S1).

Of all screened newborns, 1,348,062 were tested in 30 laboratories that performed repeat sampling when initial values were below the assay-specific cutoff, whereas the remaining 32,572 were tested in two laboratories that did not perform repeat sampling. Among newborns screened in laboratories using repeat sampling, 3,425 (0.25%) required a second dried blood spot (DBS) specimen and 687 (0.05%) were ultimately referred. The remaining 2,738 newborns had TREC/KREC values exceeding the laboratory-specific cutoffs in the second DBS specimen, and they were not referred to IEI centers. At laboratories without repeat sampling, 182 (0.56%) newborns were directly referred after initial values fell below the assay-specific cutoff. A total of 869 newborns (overall referral rate = 0.06%) were referred to IEI centers. Among the 869 referred newborns, 488 had low TREC only, 319 had low KREC only, and 62 had both low TREC and KREC. Among all screened newborns, 550 (0.04%) had low TREC values, and 381 (0.03%) had low KREC values.

### Final diagnoses and clinical course

**Figure 2.**
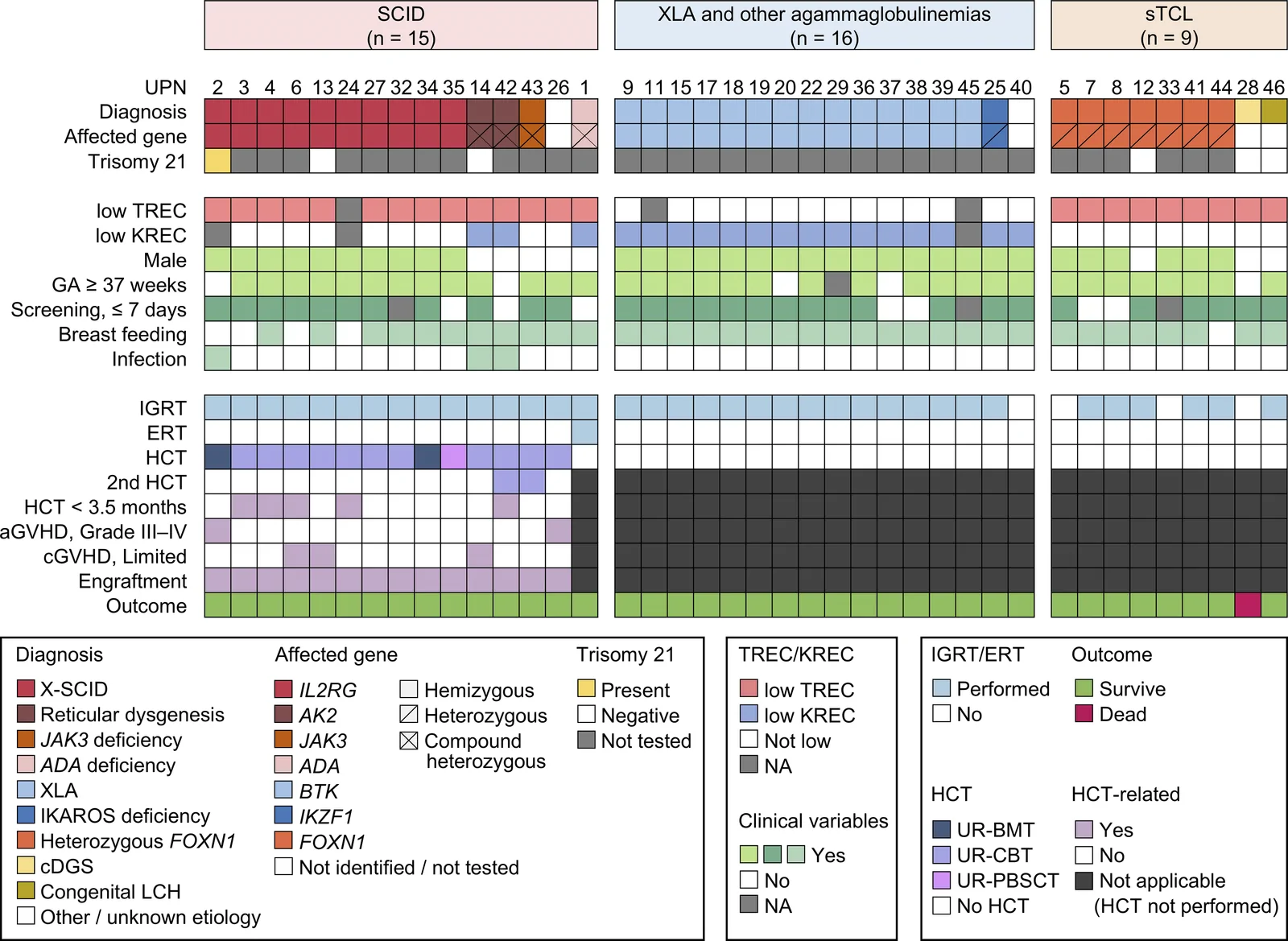
**Summary of 40 infants with IEIs identified by TREC/KREC-based NBS.** Each column represents a confirmed case with clinical characteristics and interventions. ERT, enzyme replacement therapy; NA, not available. GA, gestational age; cDGS, complete DiGeorge syndrome; LCH, Langerhans cell histiocytosis; aGVHD, acute graft-versus-host disease; cGVHD, chronic graft-versus-host disease; UR-BMT, unrelated donor bone marrow transplantation; UR-CBT, unrelated donor cord blood transplantation; UR-PBSCT, unrelated donor peripheral blood stem cell transplantation.

Diagnostic evaluations confirmed 40 cases of IEIs (Fig. 2; and Tables S3 and S4): 15 with SCID (X-linked SCID [*n* = 10], reticular dysgenesis [*n* = 2], adenosine deaminase [*ADA*] deficiency [*n* = 1], *JAK3* deficiency [*n* = 1], T^−^B^+^NK^+^ SCID [*n* = 1]), 16 with XLA and other agammaglobulinemias (XLA [*n* = 14], IKAROS deficiency [*n* = 1], unknown etiology [*n* = 1]), and 9 with sTCL (heterozygous *FOXN1* variants [*n* = 7], complete DiGeorge syndrome [*n* = 1], congenital Langerhans cell histiocytosis [*n* = 1]). Eight of these infants have been described in the literature (details and corresponding references are provided in Tables S3 and S4) (17, 30, 31, 32, 33, 34, 35, 36). The incidence of SCID was 1 in 92,042 screened newborns, and that of XLA and other agammaglobulinemias was 1 in 86,290. The positive predictive values (PPVs) for all referrals and marker-specific referrals were as follows. Among all referred infants, PPV(SCID/all referrals) was 1.7% (15/869; 95% confidence interval [CI], 1.0–2.8%), and PPV(XLA and other agammaglobulinemias/all referrals) was 1.8% (16/869; 95% CI, 1.1–3.0%). Among marker-specific referrals, PPV(SCID/TREC referrals) was 2.7% (15/550; 95% CI, 1.5–4.5%), PPV(SCID+sTCL/TREC referrals) was 4.4% (24/550; 95% CI, 2.8–6.4%), and PPV(XLA and other agammaglobulinemias/KREC referrals) was 4.2% (16/381; 95% CI, 2.4–6.7%). The median gestational age was 38 wk (interquartile range [IQR], 37–39 wk); the shortest gestational age was 29 wk in an infant with reticular dysgenesis (36).

At diagnosis, 35 infants (88%) were being breastfed. Three infants (8%) developed infections, including one with SARS-CoV-2 and two of unknown origin. IGRT was initiated in 36 infants (90%). One infant with *ADA* deficiency received enzyme replacement therapy. At last follow-up (median, 18 mo; IQR, 12–19 mo; range, 1–51 mo), 39 of the 40 infants (98%) were alive. One infant with complete DiGeorge syndrome died of circulatory failure associated with congenital heart disease.

**Figure S2.**
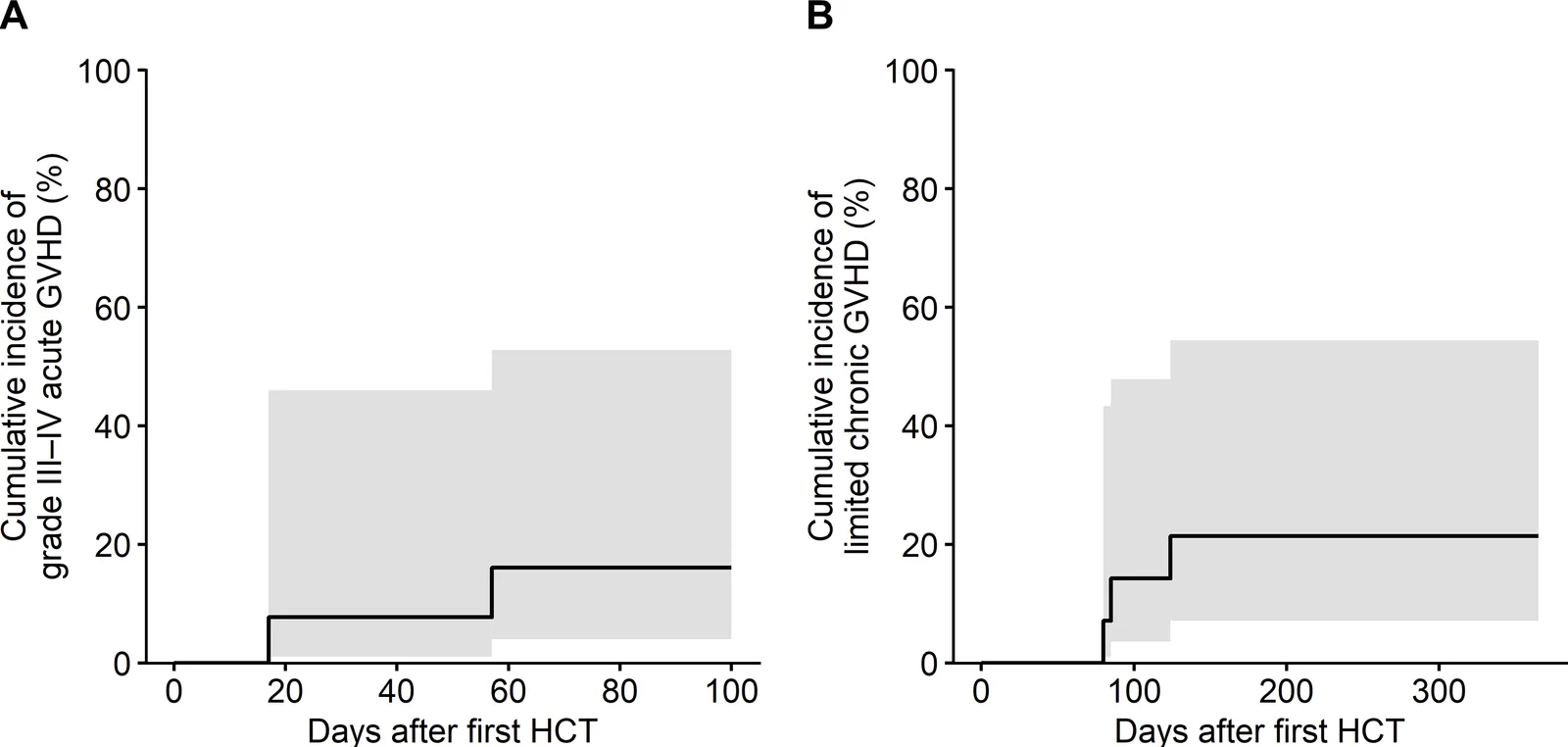
**Cumulative incidence of GVHD after first HCT. (A)** Grade III–IV acute GVHD within 100 days. **(B)** Limited chronic GVHD within 1 year. Death and second transplantation were treated as competing events. Shaded areas indicate 95% CIs.

In total, 14 infants with SCID underwent HCT, including 10 with X-SCID, 2 with reticular dysgenesis, 1 with *JAK3* deficiency, and 1 with T^−^B^+^NK^+^ SCID. Transplantation details and outcomes are summarized in Table S5. The median age at transplantation was 130 days (IQR, 105–148 days; range, 59–427 days). Five infants (36%) underwent HCT before 3.5 mo of age. Graft sources included unrelated cord blood (*n* = 11), unrelated bone marrow (*n* = 2), and peripheral blood stem cells (*n* = 1). Conditioning regimens were used in 12 infants (86%). Graft-versus-host disease (GVHD) prophylaxis comprised tacrolimus plus short-term methotrexate (*n* = 12), tacrolimus plus methotrexate and mycophenolate mofetil (*n* = 1), and tacrolimus alone (*n* = 1). The day-100 cumulative incidence of grade III–IV acute GVHD was 16.1% (95% CI, 4.0–52.8%), while the 1-year cumulative incidence of limited chronic GVHD was 21.4% (95% CI, 7.1–54.4%) (Fig. S2). 12 infants (86%) achieved sustained engraftment after the first HCT. The remaining two initially underwent unconditioned HCT to control severe infections and achieved engraftment with mixed chimerism; however, due to insufficient immune reconstitution, both underwent a second HCT and achieved sustained engraftment. Overall, sustained engraftment was achieved in all 14 infants.

### Coverage trends after implementation of the national pilot project

**Figure 3.**
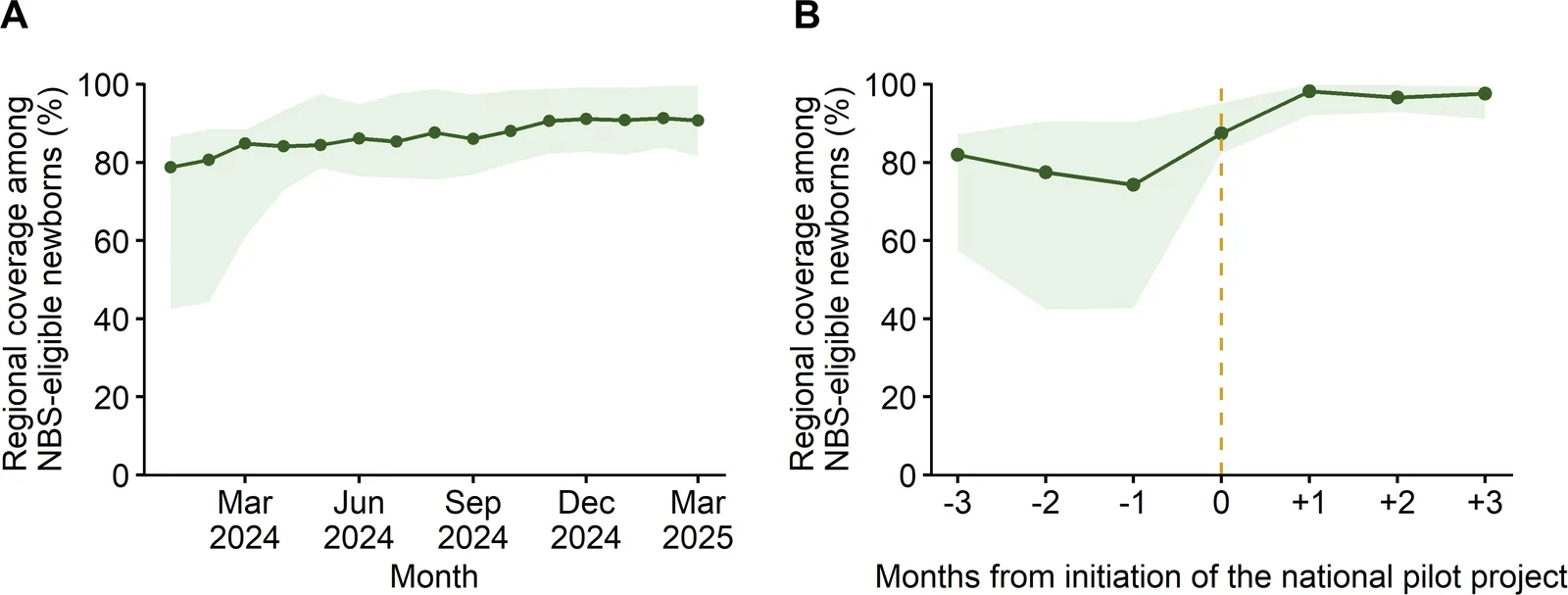
**Changes in regional coverage among NBS-eligible newborns after implementation of the national pilot project. (A)** Monthly coverage across all regions (*n* = 67; January 2024–March 2025). **(B)** Coverage before and after implementation of the national pilot project in participating regions with available monthly data (*n* = 30). Solid lines represent the median, while shaded areas indicate the IQR.

Fig. 3 A presents the monthly regional coverage among NBS-eligible newborns across all regions. During the study period, 37 of 67 regions joined the publicly funded national pilot project. Implementation began in March 2024 and proceeded sequentially across regions. Changes in coverage were evaluated in the 30 regions with complete pre- and postimplementation monthly data (Fig. 3 B). Of the 37 regions, 30 had transitioned from the optional program, under which screening was available on a self-paid or municipally subsidized basis. Seven regions had not offered TREC/KREC-based NBS under the optional program and initiated screening only after joining the publicly funded national pilot project; these regions were therefore excluded from the pre–post comparison. For the analyzed regions, coverage among NBS-eligible newborns increased from 77.1% (IQR, 42.0%–90.0%) during the optional program period to 97.7% (IQR, 92.5%–99.4%) after implementation of the national pilot project. Annual population-based coverage among all live births and regional coverage among NBS-eligible newborns are summarized in Table S6.

### Assay kit–specific variability in referral rates

**Figure 4.**
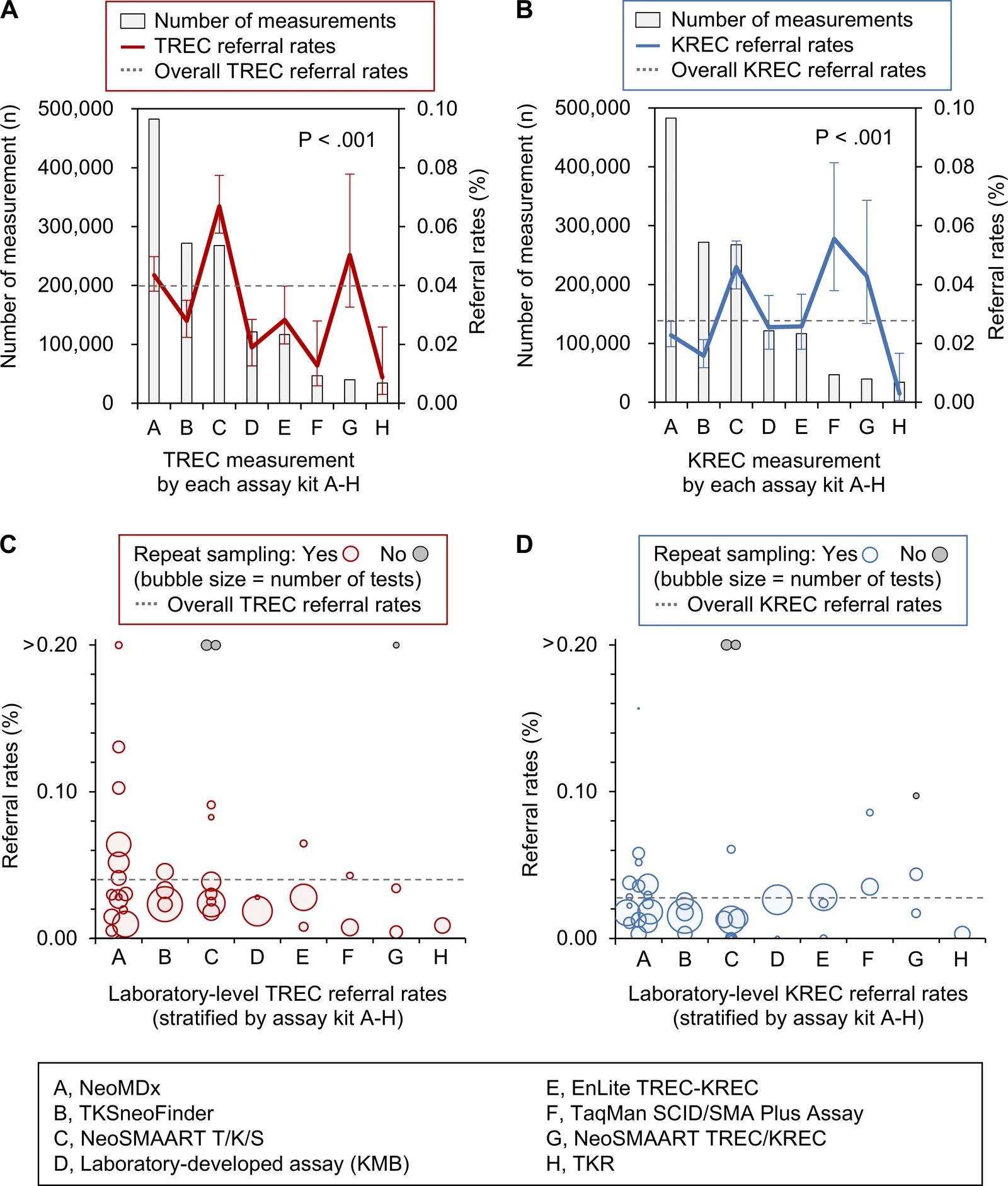
**Assay-specific referral rates and interlaboratory heterogeneity in TREC/KREC-based NBS. (A and B)** Number of tests performed and TREC/KREC referral rates for each assay kit. Solid lines indicate assay kit–specific referral rates, and dotted lines indicate the overall TREC/KREC referral rates. P values were calculated using chi-square tests comparing referral rates across assay kits. **(C and D)** Laboratory-level referral rates, stratified by the assay kit. The bubble size represents the number of tests. Gray bubbles indicate no repeat sampling. Dotted lines show overall rates (TREC 0.04%, KREC 0.03%). Values >0.20% are truncated.

Referral rates differed significantly across the assay kits, ranging from 0.01 to 0.07% for TREC (P < 0.001) and from 0.003 to 0.06% for KREC (P < 0.001) (Fig. 4, A and B). Substantial variability was also observed between laboratories using the same assay kit (Fig. 4, C and D). Three high referral rate values originated from two laboratories that did not perform repeat sampling.

**Figure 5.**
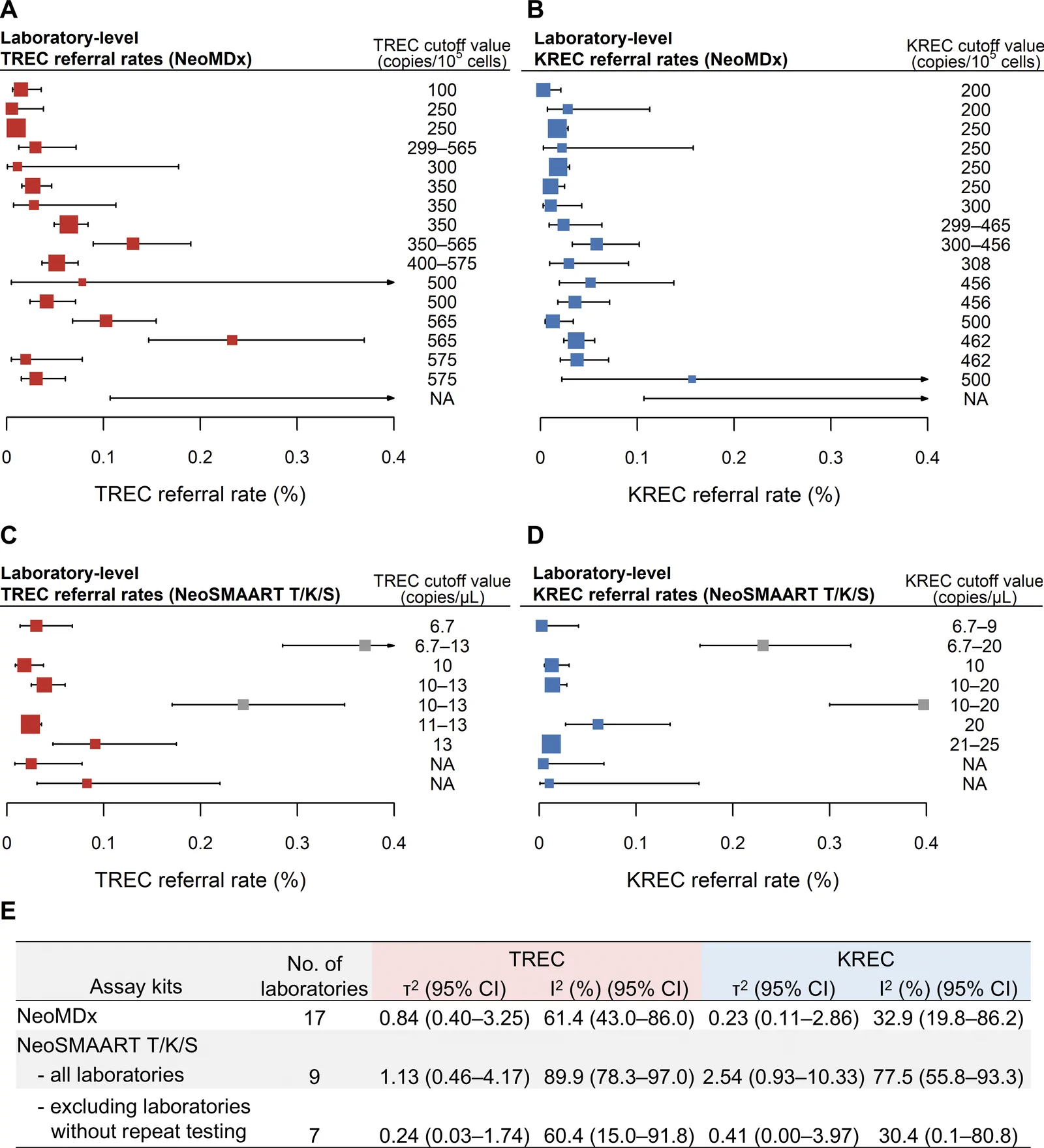
**Interlaboratory heterogeneity in TREC and KREC referral rates by the assay kit. (A–D)** Laboratory-level referral rates with 95% CIs for kits used by at least five laboratories. Each point represents individual laboratories. The point size reflects the number of tests. Laboratories are ordered by laboratory-defined cutoff values. **(E)** Heterogeneity statistics (I^2^ and τ^2^ with 95% CIs). Values >0.40% are truncated.

Interlaboratory heterogeneity was assessed for the two assay kits used by five or more laboratories, namely, NeoMDx (*n* = 17) and NeoSMAART T/K/S (*n* = 9) (Fig. 5). For both kits, referral rates were heterogeneous across laboratories, even after excluding laboratories that did not use repeat sampling. Kit-specific PPVs did not differ significantly among assays (Table S7).

## Discussion

This cohort study of over 1.3 million newborns represents the first comprehensive nationwide analysis of TREC/KREC-based NBS in Japan. The program enabled early identification of SCID and XLA, allowing timely initiation of HCT or IGRT. Our findings also demonstrate that variability in laboratory practices and funding structures can substantially influence screening performance within a nationwide but decentralized framework.

Clinical outcomes after early diagnosis were highly favorable: 39 of 40 infants with IEIs (98%) were alive at last follow-up. All 14 infants with SCID who underwent HCT achieved sustained engraftment and remained alive, and the infant with *ADA* deficiency treated with enzyme replacement therapy was also alive. The only death occurred in an infant with complete DiGeorge syndrome and was attributable to severe congenital heart disease rather than immunodeficiency-related complications. By comparison, a previous nationwide retrospective study from Japan of SCID patients treated before NBS implementation reported a 10-year overall survival of 67% after HCT (13). The uniformly favorable outcomes in our screened cohort suggest that NBS may contribute not only to earlier diagnosis but also to improved survival by enabling better pretransplant management and infection control.

Screening coverage increased substantially after implementation of the national pilot project, rising from 77.1% under the optional, self-paid framework to 97.7% following transition to publicly funded screening. This policy change created a natural experiment within a single national healthcare system, allowing us to evaluate the effect of public funding on screening coverage. Under the optional self-paid framework, total caregiver payments varied by region, facility, and bundled optional tests but generally ranged from JPY 5,000 to 15,000 (approximately USD 30–90 or EUR 27–81) per infant. Notably, this coverage increase was observed in Japan, a country generally considered socioeconomically advantaged, where a substantial proportion of eligible newborns did not undergo screening under the voluntary, self-paid framework. Previous economic evaluations have suggested that TREC-based NBS for SCID is likely to be cost-effective in high-income settings, although cost-effectiveness analyses of KREC screening remain limited (37, 38, 39). These findings suggest that even in high-income countries, population-based screening programs with clear clinical benefit may achieve near-universal coverage when publicly funded (40).

Variability in referral rates was observed across assay kits and laboratories. At the laboratory level, referral rates were markedly higher in the two laboratories that did not use repeat sampling (0.56 vs. 0.05%; Fig. 1). These findings support the need for repeat sampling in standardized TREC/KREC-based NBS algorithms to reduce unnecessary referrals. Variation also persisted between laboratories using the same assay kit, even after excluding those that did not use repeat sampling (Fig. 5). This remaining heterogeneity likely reflects differences in laboratory practices, including cutoff setting policies (1, 4, 41).

Differences in laboratory practices and funding structures may lead to regional inequities, impose greater burdens on IEI centers, and increase parental anxiety (1, 2, 9, 42). Although the overall incidence, referral rates, and PPVs observed in this program were comparable to those reported internationally, PPVs for rare disorders should be interpreted with caution. They are sensitive to small changes in case numbers and reflect the proportion of true-positive cases among referred infants, rather than the absolute number of referrals (1, 41, 43). They may also be influenced by differences in screening algorithms, cutoff settings, and repeat-sampling policies across programs. As a result, similar PPVs may still translate into different burdens for infants, families, and healthcare systems. These findings thus highlight the importance of standardized protocols. A recent global survey of TREC-based SCID NBS also documented substantial variability in analytical and postanalytical practices across screening programs (44). Harmonized screening protocols and external quality assurance are essential for improving the accuracy and consistency of TREC/KREC-based screening (2, 45).

Several limitations of this study should be acknowledged. First, participation in the NBS program was voluntary, and the national pilot project had not yet been implemented in all regions of Japan during the study period. Second, the retrospective design and relatively short follow-up limit the evaluation of long-term outcomes, including development and quality of life. Third, the present study did not include detailed data collection for newborns whose initially low TREC/KREC levels normalized on repeat sampling or for referred newborns outside the diagnostic categories analyzed in this study. Fourth, we did not evaluate the psychological and economic impacts of false-positive cases or the cost-effectiveness of the program.

In conclusion, this large nationwide cohort demonstrates that TREC/KREC-based NBS can effectively identify SCID and XLA, enabling timely intervention and favorable short-term outcomes. The increase in coverage after the introduction of publicly funded screening suggests that sustained public investment is essential for equitable access. At the same time, the interlaboratory variability highlights the need for standardized screening policies, including repeat-sampling procedures and cutoff setting practices, alongside centralized quality assurance. These findings support the integration of TREC/KREC-based testing into a universal national NBS program and may inform international efforts toward more harmonized implementation.

## Materials and methods

### Study population

The study population comprised 1,380,634 newborns who underwent TREC/KREC screening in Japan between April 1, 2020, and March 31, 2025. Screening was conducted under two frameworks: the optional program and the national pilot project. Under the optional program, screening was offered on a self-paid or municipally subsidized basis. For the national pilot project, screening was publicly funded with no out-of-pocket payments. The national pilot project was introduced sequentially across participating regions beginning in March 2024.

For analysis, data were aggregated at the level of administrative units, which were defined as prefectures and designated cities (*n* = 67). Of the 35 laboratories that perform the standard publicly funded NBS for inborn errors of metabolism in Japan, 32 also performed TREC/KREC screening across the 67 administrative units, while the remaining three only performed metabolic NBS.

### TREC/KREC assessment

TREC/KREC levels were quantified from DBS specimens, typically collected 4–6 days after birth. Each of the 32 NBS laboratories independently employed either commercial or laboratory-developed assays and applied laboratory-specific cutoff values (Table S1). Several laboratories changed assay kits or revised cutoff values during the study period. Assays used included EnLite TREC/KREC (Revvity); NeoMDx (Revvity); NeoSMAART TREC/KREC (Sekisui Medical); NeoSMAART T/K/S (Sekisui Medical); TaqMan SCID/SMA Plus Assay (Thermo Fisher Scientific); TKR (Nihon Techno Service Co., Ltd.); TKSneoFinder (Shimadzu SDC); and a laboratory-developed assay used at KM Biologics Co., Ltd. Genomic DNA was extracted from DBS specimens. TREC/KREC levels were measured by real-time polymerase chain reaction according to the manufacturers’ instructions.

### Screening algorithm

Samples with TREC/KREC levels above the cutoff were considered screen-negative. Samples with levels below the cutoff underwent repeat testing using a second DBS specimen. Newborns with persistently low values were referred to designated IEI centers for comprehensive immunological evaluations. Repeat sampling was used by 30 of the 32 laboratories, whereas the remaining two laboratories directly referred newborns with low initial TREC/KREC values. The referred newborns were categorized according to the marker pattern as follows: low TREC only, when only TREC levels were below the cutoff; low KREC only, when only KREC levels were below the cutoff; and both low TREC and KREC, when both markers were below the cutoff.

### Data collection

Questionnaires were distributed to the 32 NBS laboratories and 69 specialized IEI centers nationwide. Laboratories provided information on the assay type, total number of newborns screened (under the optional program or national pilot project), and the number of infants requiring repeat testing or referral. IEI centers provided detailed clinical information for patients included in the study, including demographic characteristics, breastfeeding history, infectious complications, genetic and cytogenetic findings, TREC/KREC values, blood counts, immunoglobulin levels, lymphocyte subsets, use of IGRT, use of enzyme replacement therapy, and survival outcomes. For analysis, confirmed cases were categorized into three groups: (1) SCID, (2) XLA and other agammaglobulinemias, and (3) sTCL. In this study, SCID referred to classical SCID, whereas sTCL was analyzed separately as a secondary finding. Other atypical forms of combined immunodeficiency were outside the scope of this study. For patients who underwent HCT, additional data were collected on age at transplantation, graft source, human leukocyte antigen compatibility, conditioning regimen, GVHD prophylaxis, GVHD onset and severity, engraftment, and second transplantation.

### Coverage definitions

We defined two coverage measures. Population-based coverage was defined as the proportion of all live births in Japan that underwent TREC/KREC-based NBS during the study period. Regional coverage among NBS-eligible newborns was defined within each region as the proportion of newborns who underwent TREC/KREC-based NBS among those who received the existing standard publicly funded NBS program for inborn errors of metabolism.

For pre- and postimplementation comparisons after the rollout of the publicly funded TREC/KREC screening, analyses were restricted to regions participating in the national pilot project with available monthly data spanning the rollout period. Within each region, coverage was summarized as the median monthly value during the 3 mo immediately before and after the rollout. These region-specific coverage estimates were then summarized across regions, and the distribution was reported as the median and IQR.

### Statistical analysis

The primary analyses focused on overall referral rates and PPVs. Secondary analyses included comparisons across assay kits and assessments of interlaboratory heterogeneity. Posttransplant outcomes were reported using descriptive statistics; the cumulative incidence of acute and chronic GVHD was estimated using the Fine–Gray method, with 95% CIs. For GVHD analyses, death and second transplantation were treated as competing events.

Referral rates were calculated as the number of referred infants divided by the total number of screened infants. PPVs were calculated using two approaches. For international comparison, PPV(SCID/all referrals) and PPV(XLA and other agammaglobulinemias/all referrals) were calculated as the proportions of infants diagnosed with SCID and XLA or other agammaglobulinemias among all referred infants, respectively. To evaluate marker-specific screening performance, PPV(SCID/TREC referrals), PPV(SCID+sTCL/TREC referrals), and PPV(XLA and other agammaglobulinemias/KREC referrals) were calculated as the proportion of infants with SCID among low-TREC referrals, the proportion of infants with SCID plus sTCL among low-TREC referrals, and the proportion of infants with XLA or other agammaglobulinemias among low-KREC referrals, respectively. Referral rates were compared across kits using the chi-square test. The 95% CIs for referral rates and PPVs were estimated using the Wilson method. For kit-level comparisons, kit-specific PPV(SCID/TREC referrals) and kit-specific PPV(XLA and other agammaglobulinemias/KREC referrals) were calculated as the proportion of confirmed cases among marker-specific referrals within each assay kit. Interlaboratory variability within each assay kit was assessed using random-effects models fitted with restricted maximum likelihood. Heterogeneity was quantified using the τ^2^ and I^2^ statistics with 95% CIs. Because I^2^ estimates are unstable when based on small numbers, values were only calculated for assay kits used by five or more laboratories. Statistical analyses were conducted using EZR (Saitama Medical Center, Jichi Medical University), a graphical user interface for R (The R Foundation for Statistical Computing) (46). Random-effects models and forest plots were generated using the metafor package (version 3.8–1) implemented in R version 4.5.1 (2025-06-13 ucrt). A two-sided P value <0.05 was considered statistically significant.

### Literature-based international comparison of PPVs

For international comparisons, we searched PubMed for NBS studies measuring both TREC and KREC published up to February 28, 2026; for each country, the most recent report was used. 11 studies met these criteria and provided sufficient data to allow recalculation of PPV(SCID/all referrals) and PPV(XLA and other agammaglobulinemias/all referrals) (Table S2). To ensure comparability across studies, PPVs were recalculated using harmonized diagnostic criteria. For SCID, PPVs were recalculated using a classical SCID definition, excluding leaky SCID, combined immunodeficiency, and other causes of sTCL such as complete DiGeorge syndrome, even when classified as SCID in the original reports. For XLA and other agammaglobulinemias, secondary causes of hypogammaglobulinemia were excluded.

### Online supplemental material

Supplemental material includes Tables S1, S2, S3, S4, S5, S6, S7, and S8; and Fig. S1 shows annual population-based coverage of TREC/KREC-based NBS in Japan. Fig. S2 shows the cumulative incidence of GVHD after first HCT. Table S1 shows cutoff values for TREC/KREC screening assays among participating laboratories during the study period. Table S2 shows international comparison of TREC/KREC NBS programs. Table S3 shows genetic information of infants identified through TREC/KREC NBS. Table S4 shows clinical and immunological characteristics at diagnosis. Table S5 shows details of HCT. Table S6 shows annual coverage of TREC/KREC-based NBS in Japan. Table S7 shows PPVs for TREC/KREC screening assays. Table S8 shows lists of surveyed institutions and NBS laboratories.

## Ethics statement

This study was approved by the Institutional Review Board of Nagoya University Graduate School of Medicine (Approval No. 2023-0413) and conducted in accordance with the Declaration of Helsinki. Informed consent was obtained through an opt-out process approved by the Institutional Review Board.

## Supplementary Material

Table S1 shows cutoff values for TREC/KREC screening assays among participating laboratories during the study period.

Table S2 shows international comparison of TREC/KREC NBS programs.

Table S3 shows genetic information of infants identified through TREC/KREC NBS.

Table S4 shows clinical and immunological characteristics at diagnosis.

Table S5 shows details of HCT.

Table S6 shows annual coverage of TREC/KREC-based NBS in Japan.

Table S7 shows PPVs for TREC/KREC screening assays.

Table S8 shows lists of surveyed institutions and NBS laboratories.

## Data Availability

The data are available from the corresponding author upon reasonable request. Requests for additional patient-level genetic data and accession information will be considered individually through the corresponding author.
