# Supplementary material for: Nationwide TREC/KREC-based newborn screening and clinical outcomes in Japan
Source: J Hum Immun. 2026 Aug 17;2(5):e20260083. doi: 10.70962/jhi.20260083 (PMC13521547; doi:10.70962/jhi.20260083)
Supplement: Supplementary file 1 [file jhi_20260083_tables1.docx]

**Table S1. Cutoff values for TREC/KREC screening assays among participating laboratories during the study period**

| Assay kits (Manufacturer) | Cutoff range | | Unit of TREC/KREC values |
| --- | --- | --- | --- |
|  | TREC | KREC |  |
| EnLite TREC-KREC (Revvity, USA) | 30 | 20–30 | copies/μL |
| NeoMDx (Revvity, USA) | 100–575 | 100–500 | copies/10^5^ cells |
| NeoSMAART TREC/KREC (Sekisui Medical, Japan) | 6.7–30 | 6.7–30 | copies/μL |
| NeoSMAART T/K/S (Sekisui Medical, Japan) | 6.7–20 | 6–25 | copies/μL |
| TaqMan SCID/SMA Plus Assay (Thermo Fisher Scientific, USA) | 20 | 20 | copies/μL |
| TKR (Nihon Techno Service Co., Ltd., Japan) | 15–20 | 10–20 | copies/μL |
| TKSneoFinder (Shimadzu SDC, Japan) | 9–20 | 7–20 | copies/μL |
| A laboratory-developed assay (KM Biologics Co., Ltd., Japan) | 30 | 10 | copies/μL |

KREC, kappa-deleting recombination excision circle; TREC, T-cell receptor excision circle.
