# Supplementary material for: Nationwide TREC/KREC-based newborn screening and clinical outcomes in Japan
Source: J Hum Immun. 2026 Aug 17;2(5):e20260083. doi: 10.70962/jhi.20260083 (PMC13521547; doi:10.70962/jhi.20260083)
Supplement: Supplementary file 2 [file jhi_20260083_tables2.docx]

**Table S2. International comparison of TREC/KREC newborn screening programs**

| Country (Region) | Study Period | Newborns (n) | Referral rates | SCID | | | XLA and other  agammaglobulinemias | | | Ref |
| --- | --- | --- | --- | --- | --- | --- | --- | --- | --- | --- |
|  |  |  |  | (n) | Incidence | PPV(SCID/all referrals) | (n) | Incidence | PPV(XLA and other  agammaglobulinemias /all referrals) |  |
| Russia | 2023–2024 | 2,348,872 | 0.03% | 37 | 1 : 63,483 | 5.3% | 49 | 1 : 47,936 | 7.0% | (S1) |
| Japan | 2020–2025 | 1,380,634 | 0.06% | 15 | 1 : 92,042 | 1.7% | 16 | 1 : 86,290 | 1.8% | This study |
| Switzerland | 2019–2023 | 435,985 | 0.02% | 10 | 1 : 43,599 | 9.7% | 8 | 1 : 54,498 | 7.8% | (S2) |
| Ukraine | 2022–2025 | 398,415 | 0.01% | 5 | 1 : 79,683 | 8.8% | 7 | 1 : 56,916 | 12.3% | (S3) |
| Czechia | 2022–2023 | 198,675 | 0.03% | 1 | 1 : 198,675 | 1.7% | 10 | 1 : 19,868 | 17.2% | (S4) |
| China (Zhejiang) | 2021–2022 | 103,240 | 0.11% | 1 | 1 : 103,240 | 0.9% | 2 | 1 : 51,620 | 1.8% | (S5) |
| Italy (Tuscany) | 2018–2022 | 94,319 | 0.10% | 2 | 1 : 47,160 | 2.1% | 0 | - | - | (S6) |
| Sweden (Stockholm) | 2013–2016 | 89,462 | 0.10% | 2 | 1 : 44,731 | 2.2% | 0 | - | - | (S7, S8) |
| Poland and Germany | 2018–2019 | 44,287 | 0.02% | 1 | 1 : 44,287 | 12.5% | 1 | 1 : 44,287 | 12.5% | (S9) |
| Spain (Andalusia) | 2021–2022 | 14,035 | NA | 0 | - | - | 0 | - | - | (S10) |
| Bulgaria (Sofia) | 2021 | 2,228 | NA | 0 | - | - | 0 | - | - | (S11) |
| Turkey (Ankara) | 2015–2016 | 1,960 | NA | 0 | - | - | 0 | - | - | (S12) |
| Colombia (Medellin) | NA | 1,092 | NA | 0 | - | - | 0 | - | - | (S13) |

NA, not available; PPV, positive predictive value; SCID, severe combined immunodeficiency; XLA, X-linked agammaglobulinemia.

**Supplemental References**

S1.	Marakhonov, A., A. Mukhina, I. Efimova, N. Balinova, M. Ampleeva, A. Bobreshova, Y. Rodina, D. Pershin, V. Zabnenkova, O. Ryzhkova, Z. Markova, N. Shilova, I. Zhanin, K. Savostyanov, S. Matulevich, F. Bilalov, A. Koroteev, A. Donnikov, D. Trofimov, T. Bairova, G. Seitova, S. Mordanov, E. Nikolaeva, Z. Esmurzieva, E. Skorobogatova, L. Olkhova, L. Vakhonina, D. Kostenko, G. Bronin, S. Zimin, T. Bykova, D. Balashov, R. Zinchenko, N. Grachev, S. Voronin, A. Shcherbina, and S. Kutsev. 2026. First 2-year experience of nationwide newborn screening for severe forms of T and B cell immunodeficiency: 2.3 million newborns analyzed using TREC and KREC in Russia. *Front Immunol*. 17:1742811. doi:10.3389/fimmu.2026.1742811.

S2.	Soomann, M., S. Prader, S. Sluka, C. Boucq, J. Pachlopnik Schmid, J. Truck, and R.S.N.B.S.G. Swiss. 2026. Five Years of Combined Newborn Screening Quantifying TREC and KREC in Switzerland. *J Allergy Clin Immunol Pract*. 14:243-252. doi:10.1016/j.jaip.2025.10.031.

S3.	Boyarchuk, O., H. Makukh, A. Volokha, A. Bondarenko, N. Mytsyk, O. Barvinska, I. Shymanska, Y. Pohuliai, M. Veropotvelyan, A. Haviley, M. Hurina, Y. Romanyshyn, M. Trophymova, I. Hrabovska, O. Malko, T. Tsanko, O. Tykholaz, O. Lysytsia, N. Samonenko, and N. Olkhovych. 2025. Newborn screening for SCID and severe T- and B-cell lymphopenia in Ukraine: the first analysis of the results, 2022-2025. *Front Immunol*. 16:1709657. doi:10.3389/fimmu.2025.1709657.

S4.	Bloomfield, M., E. Hlavackova, H. Schneiderova, M. Turnovec, L. Tichy, Z. Cech, P. Chrastina, L. Dvorakova, K. Peskova, R. Formankova, P. Riha, M. Vlkova, P. Bejdak, M. Havlisova, E. Fronkova, T. Kalina, V. Bily, D. Ricna, H. Grombirikova, P. Sedlacek, J. Litzman, T. Freiberger, A. Sediva, and A. Klocperk. 2025. Beyond TREC: Pivotal role of tandem TREC/KREC assay in Czech SCID NBS pilot programme. *Pediatr Allergy Immunol*. 36:e70100. doi:10.1111/pai.70100.

S5.	Chen, C., C. Zhang, D.W. Wu, B.Y. Wang, R. Xiao, X.L. Huang, X. Yang, Z.G. Gao, and R.L. Yang. 2024. Comprehensive newborn screening for severe combined immunodeficiency, X-linked agammaglobulinemia, and spinal muscular atrophy: the Chinese experience. *World J Pediatr*. 20:1270-1282. doi:10.1007/s12519-024-00846-7.

S6.	Ricci, S., V. Guarnieri, F. Capitanini, C. Pelosi, V. Astorino, S. Boscia, E. Calistri, C. Canessa, M. Cortimiglia, F. Lippi, L. Lodi, S. Malvagia, M. Moriondo, G. La Marca, and C. Azzari. 2024. Expanded Newborn Screening for Inborn Errors of Immunity: The Experience of Tuscany. *J Allergy Clin Immunol Pract*. 12:1622-1630. doi:10.1016/j.jaip.2024.04.011.

S7.	Barbaro, M., A. Ohlsson, S. Borte, S. Jonsson, R.H. Zetterstrom, J. King, J. Winiarski, U. von Dobeln, and L. Hammarstrom. 2017. Newborn Screening for Severe Primary Immunodeficiency Diseases in Sweden-a 2-Year Pilot TREC and KREC Screening Study. *J Clin Immunol*. 37:51-60. doi:10.1007/s10875-016-0347-5.

S8.	Zetterström, R.H., M. Barbaro, A. Ohlsson, S. Borte, S. Jonsson, J. Winiarski, U. Von Döbeln, and L. Hammarström. 2017. Newborn Screening for Primary Immune Deficiencies with a TREC/KREC/ACTB Triplex Assay—A Three-Year Pilot Study in Sweden. *Int J Neonatal Screen*. 3:11. doi:10.3390/ijns3020011.

S9.	Gizewska, M., K. Durda, T. Winter, I. Ostrowska, M. Oltarzewski, J. Klein, O. Blankenstein, H. Romanowska, E. Krzywinska-Zdeb, M.F. Patalan, E. Bartkowiak, N. Szczerba, S. Seiberling, B. Birkenfeld, M. Nauck, H. von Bernuth, C. Meisel, E.A. Bernatowska, M. Walczak, and M. Pac. 2020. Newborn Screening for SCID and Other Severe Primary Immunodeficiency in the Polish-German Transborder Area: Experience From the First 14 Months of Collaboration. *Front Immunol*. 11:1948. doi:10.3389/fimmu.2020.01948.

S10.	De Felipe, B., C. Delgado-Pecellin, M. Lopez-Lobato, P. Olbrich, P. Blanco-Lobo, J. Marquez-Fernandez, C. Salamanca, B. Mendoza, R. Castro-Serrano, C. Duque, M. Moreno-Prieto, M. Madruga-Garrido, J.M. Lucena, R.M. Fernandez, M. Ruiz-Camacho, A. Varona, and O. Neth. 2025. Neonatal Screening for Spinal Muscular Atrophy and Severe T- and B-Cell Lymphopenias in Andalusia: A Prospective Study. *Int J Neonatal Screen*. 11:11. doi:10.3390/ijns11010011.

S11.	Marinova, M., A. Georgyeva, V. Yordanova, N. Ivanov, V. Atanasova, E. Naumova, and S.M. Kandilarova. 2022. Implementation of TREC/KREC detection protocol for newborn SCID screening in Bulgaria: a pilot study. *Cent Eur J Immunol*. 47:339-349. doi:10.5114/ceji.2022.124396.

S12.	Kutlug, S., M. Karadag Alpaslan, G. Hancioglu, S. Elif Ozyazici Ozkan, D. Cemile Yesilirmak, H. Bulut, C. Aygun, G. Ogur, and A. Yildiran. 2021. Multiplex PCR-Based Newborn Screening for Severe T and B-Cell Lymphopenia: The first Pilot Study in Turkey. *Sisli Etfal Hastan Tip Bul*. 55:551-559. doi:10.14744/SEMB.2020.09623.

S13.	Gutierrez-Hincapie, S., C. Muskus-Lopez, I.P. Sanchez, J.L. Franco-Restrepo, and C.M. Trujillo-Vargas. 2024. First pilot study for newborn screening of severe T and B lymphopenias in Colombia. *Biomedica*. 44:94-106. doi:10.7705/biomedica.7568.
