# Supplementary material for: Nationwide TREC/KREC-based newborn screening and clinical outcomes in Japan
Source: J Hum Immun. 2026 Aug 17;2(5):e20260083. doi: 10.70962/jhi.20260083 (PMC13521547; doi:10.70962/jhi.20260083)
Supplement: Supplementary file 3 [file jhi_20260083_tables3.docx]

**Table S3. Genetic information of infants identified through TREC/KREC newborn screening**

| UPN | Diagnosis | Gene | Variants | Zygosity | Ref |
| --- | --- | --- | --- | --- | --- |
| 2 | X-SCID | *IL2RG* | c.455-2A>G | Hemizygous | (S14) |
| 3 | X-SCID | *IL2RG* | c.318_324del, p.Phe107Lysfs*38 | Hemizygous |  |
| 4 | X-SCID | *IL2RG* | c.468T>A, p.Ile153Asn | Hemizygous |  |
| 6 | X-SCID | *IL2RG* | c.256A>C, p.Thr86Pro | Hemizygous | (S15) |
| 13 | X-SCID | *IL2RG* | c.222G>A, p.Trp74* | Hemizygous | (S16) |
| 24 | X-SCID | *IL2RG* | c.202G>T, p.Glu68* | Hemizygous |  |
| 27 | X-SCID | *IL2RG* | c.854+4A>G | Hemizygous |  |
| 32 | X-SCID | *IL2RG* | c.664C>T, p.Arg222Cys | Hemizygous |  |
| 34 | X-SCID | *IL2RG* | c.964C>T, p.Gln322* | Hemizygous |  |
| 35 | X-SCID | *IL2RG* | c.930G>A, p.Trp310* | Hemizygous |  |
| 14 | RD | *AK2* | c.498+1G>A,  c.409C>T, p.Arg137* | Compound heterozygous | (S16, S17) |
| 42 | RD | *AK2* | c.307C>T, p.Arg103Trp, c.308G>A, p.Arg103Gln | Compound heterozygous | (S18) |
| 43 | *JAK3* deficiency | *JAK3* | c.1205G>A, p.Arg402His, c.2134G>A, p.Gly712Ser | Compound heterozygous |  |
| 26 | T^-^B^+^NK^+^ SCID | - | - | - |  |
| 1 | *ADA* deficiency | *ADA* | c.881C>T, p.Thr294Ile,  c.704G>A, p.Arg235Gln | Compound heterozygous |  |
| 9 | XLA | *BTK* | c.1928C>T, p.Thr643Ile | Hemizygous |  |
| 11 | XLA | *BTK* | c.908G>A, p.Gly303Asp | Hemizygous |  |
| 15 | XLA | *BTK* | c.1868C>T, p.Ser623Leu | Hemizygous |  |
| 17 | XLA | *BTK* | del:ex6-19 | Hemizygous | (S19) |
| 18 | XLA | *BTK* | c.1696C>T, p.Pro566Ser | Hemizygous | (S20) |
| 19 | XLA | *BTK* | c.215dup, p.Asn72Lysfs*13 | Hemizygous |  |
| 20 | XLA | *BTK* | c.82C>T, p.Arg28Cys | Hemizygous |  |
| 22 | XLA | *BTK* | c.700_701del, p.Gln234Alafs*5 | Hemizygous |  |
| 29 | XLA | *BTK* | c.41C>T, p.Ser14Phe | Hemizygous |  |
| 36 | XLA | *BTK* | c.1574G>A, p.Arg525Gln | Hemizygous |  |
| 37 | XLA | *BTK* | c.119A>G, p.Tyr40Cys | Hemizygous |  |
| 38 | XLA | *BTK* | c.974+5G>A | Hemizygous |  |
| 39 | XLA | *BTK* | c.1527G>A, p.Met509Ile | Hemizygous |  |
| 45 | XLA | *BTK* | c.1574G>A, p.Arg525Gln | Hemizygous |  |
| 25 | IKAROS deficiency | *IKZF1* | c.557A>T, p.Asp186Val | Heterozygous |  |
| 40 | Agammaglobulinemia | - | - | - |  |
| 5 | Heterozygous *FOXN1* | *FOXN1* | c.958C>T, p.Arg320Trp | Heterozygous |  |
| 7 | Heterozygous *FOXN1* | *FOXN1* | c.968T>G, p.Leu323Arg | Heterozygous |  |
| 8 | Heterozygous *FOXN1* | *FOXN1* | c.968T>G, p.Leu323Arg | Heterozygous |  |
| 12 | Heterozygous *FOXN1* | *FOXN1* | c.974T>A, p.Leu325His | Heterozygous | (S16) |
| 33 | Heterozygous *FOXN1* | *FOXN1* | c.166C>A, p.Pro56Thr, c.902T>C, p.Met301Thr | Heterozygous  (two variants detected) |  |
| 44 | Heterozygous *FOXN1* | *FOXN1* | c.1315del, p.Leu439Cysfs*111 | Heterozygous |  |
| 41 | Heterozygous *FOXN1* | *FOXN1* | c.961C>T, p.His321Tyr | Heterozygous |  |
| 28 | cDGS | - | - | - |  |
| 46 | congenital LCH | - | - | - | (S21) |

ADA deficiency, adenosine deaminase deficiency; cDGS, complete DiGeorge syndrome; LCH, Langerhans cell histiocytosis; RD, reticular dysgenesis; UPN, unique patient number; X-SCID, X-linked severe combined immunodeficiency; XLA, X-linked agammaglobulinemia

**Supplemental References**

S14.	Imanaka, Y., T. Asano, K. Noma, F. Sakura, N. Nishioka, Y. Mizoguchi, T. Kamiya, H. Kanegane, and S. Okada. 2025. X-linked severe combined immunodeficiency with Down syndrome identified by newborn screening. *J Hum Immunity*. 1:e20250057. doi:10.70962/jhi.20250057.

S15.	Beppu, S., T. Nishikawa, D. Tomomasa, A. Hijikata, H. Kasabata, H. Terazono, K. Ikawa, T. Nakamura, S. Horikawa, J. Nagahama, A. Nakamura, T. Abematsu, S. Nakagawa, K. Oketani, H. Kanegane, and Y. Okamoto. 2024. Perspectives in newborn screening for SCID in Japan. Case report: newborn screening identified X-linked severe combined immunodeficiency with a novel IL2RG variant. *Front Immunol*. 15:1478411. doi:10.3389/fimmu.2024.1478411.

S16.	Wakamatsu, M., D. Kojima, H. Muramatsu, Y. Okuno, S. Kataoka, F. Nakamura, Y. Sakai, I. Tsuge, T. Ito, K. Ueda, A. Saito, E. Morihana, Y. Ito, N. Ohashi, M. Tanaka, T. Tanaka, S. Kojima, Y. Nakajima, T. Ito, and Y. Takahashi. 2022. TREC/KREC Newborn Screening followed by Next-Generation Sequencing for Severe Combined Immunodeficiency in Japan. *J Clin Immunol*. 42:1696-1707. doi:10.1007/s10875-022-01335-0.

S17.	Wakamatsu, M., Y. Kitagawa, Y. Tsumura, Y. Okuno, Y. Sato, Y. Kamachi, R. Maemura, M. Imaya, A. Yamamori, K. Narita, S. Kataoka, A. Narita, N. Nishio, S. Kojima, Y. Takahashi, M.K. Saito, and H. Muramatsu. 2026. Response to recombinant human granulocyte colony-stimulating factor in reticular dysgenesis. *J Hum Immunity*. 2:e20250232. doi:10.70962/jhi.20250232.

S18.	Kinoshita, K., H. Muramatsu, M. Wakamatsu, K. Ueda, Y. Sato, and Y. Takahashi. 2026. Reticular Dysgenesis in an Extremely Low Birth Weight Infant: A Case Report. *J Clin Immunol*. 46:53. doi:10.1007/s10875-026-02029-7.

S19.	Kinoshita, Y., J. Kido, T. Sawada, K. Sugawara, F. Nozaki, T. Mizukami, M. Nishimura, S. Yoshida, R. Tsuru, and K. Nakamura. 2025. T-Cell Receptor Excision Circle/Kappa-Deleting Recombination Excision Circle-Based Newborn Screening Program for Severe Combined Immunodeficiency in Kumamoto, Japan. *Cell Biochem Biophys*. 84:549–560. doi:10.1007/s12013-025-01873-5.

S20.	Kimizu, T., M. Nozaki, Y. Okada, A. Sawada, M. Morisaki, H. Fujita, A. Irie, K. Matsuda, Y. Hasegawa, E. Nishi, N. Okamoto, M. Kawai, K. Imai, Y. Suzuki, K. Wada, N. Mitsuda, and S. Ida. 2024. Multiplex Real-Time PCR-Based Newborn Screening for Severe Primary Immunodeficiency and Spinal Muscular Atrophy in Osaka, Japan: Our Results after 3 Years. *Genes (Basel)*. 15:314. doi:10.3390/genes15030314.

S21.	Uejima, Y., M. Iijima, K. Oshima, K. Kawabata, A. Nitta, T. Sato, Y. Sato, Z. Tamaki, T. Hosokawa, M. Shimizu, K. Koh, and A. Nakazawa. 2025. A case of early diagnosis of congenital multisystem langerhans cell histiocytosis with thymic involvement triggered by newborn screening. *Immunol Med*. 48:407-410. doi:10.1080/25785826.2025.2519725.
