# Supplementary material for: Nationwide TREC/KREC-based newborn screening and clinical outcomes in Japan
Source: J Hum Immun. 2026 Aug 17;2(5):e20260083. doi: 10.70962/jhi.20260083 (PMC13521547; doi:10.70962/jhi.20260083)
Supplement: Supplementary file 4 [file jhi_20260083_tables4.docx]

**Table S4. Clinical and immunological characteristics at diagnosis**

| UPN | Diagnosis | Gestation  (week) | TREC | KREC | Unit of  TREC/KREC values | ANC  (×10^9^/L) | ALC  (×10^9^/L) | CD3^+^  (×10^9^/L) | CD4^+^  (×10^9^/L) | CD8^+^  (×10^9^/L) | CD19^+^  (×10^9^/L) | CD45RA^+^  (×10^9^/L) | IgG  (g/L) |
| --- | --- | --- | --- | --- | --- | --- | --- | --- | --- | --- | --- | --- | --- |
| 2 | X-SCID | 35 | 4 | NA | copies/μL | 0.8 | 0.9 | 0.01 | 0.02 | 0.0 | 0.8 | 0.002 | 8.4 |
| 3 | X-SCID | 38 | 0 | 3,181 | copies/10^5^ cells | 6.2 | 0.6 | 0.0 | 0.0 | 0.0 | 0.6 | NA | 8.1 |
| 4 | X-SCID | 38 | 0 | 5,064 | copies/10^5^ cells | 5.4 | 1.3 | 0.0 | 0.0 | 0.0 | NA | NA | 9.6 |
| 6 | X-SCID | 38 | 0 | 4,473 | copies/10^5^ cells | 3.3 | 1.4 | 0.0 | 0.00 | 0.0 | 1.4 | 0.0 | NA |
| 13 | X-SCID | 39 | 4 | 307 | copies/μL | 4.5 | 1.9 | 0.05 | 0.01 | 0.001 | 1.6 | 0.01 | NA |
| 24 | X-SCID | 37 | NA | NA |  | 8.1 | 0.3 | 0.05 | 0.003 | 0.04 | NA | 0.0 | NA |
| 27 | X-SCID | 38 | 0 | 45 | copies/μL | 4.2 | 1.0 | 0.05 | 0.001 | 0.003 | 0.8 | 0.0 | 9.8 |
| 32 | X-SCID | 38 | 6.2 | 110.3 | copies/μL | 6.2 | 1.5 | 0.2 | 0.2 | 0.03 | 0.3 | NA | 6.8 |
| 34 | X-SCID | 37 | 0 | 165.5 | copies/μL | 3.1 | 0.5 | 0.001 | 0.0 | 0.0 | 0.6 | 0.0 | 7.1 |
| 35 | X-SCID | 39 | 0 | 96.1 | copies/μL | 2.4 | 0.4 | 0.02 | 0.0 | 0.0 | 0.4 | 0.0 | 9.8 |
| 14 | RD | 38 | 0 | 0 | copies/μL | 0.0 | 0.1 | 0.06 | 0.04 | 0.0 | 0.0 | 0.0 | 9.0 |
| 42 | RD | 29 | 0 | 0 | copies/10^5^ cells | 0.0 | 0.0 | 0.0 | 0.0 | 0.0 | 0.0 | 0.0 | 4.4 |
| 43 | *JAK3* deficiency | 40 | 0 | 4,042 | copies/10^5^ cells | 3.1 | 1.0 | 0.03 | 0.001 | 0.004 | 0.9 | 0.002 | 10.7 |
| 26 | T^-^B^+^NK^+^ SCID | 40 | 0 | 16 | copies/μL | 6.2 | 1.7 | 0.07 | 0.05 | 0.006 | 0.8 | 0.03 | 7.9 |
| 1 | *ADA* deficiency | 38 | 0 | 0 | copies/μL | 0.6 | 0.03 | 0.0 | 0.0 | 0.0 | 0.0 | NA | 5.9 |
| 9 | XLA | 40 | 2,939 | 206 | copies/10^5^ cells | 2.2 | 3.7 | 3.0 | 1.7 | 1.2 | 0.04 | NA | 7.0 |
| 11 | XLA | 39 | NA | 11.7 | copies/μL | 2.3 | 5.5 | 5.1 | 4.0 | 1.0 | 0.06 | NA | 5.4 |
| 15 | XLA | 37 | 2,907 | 0 | copies/10^5^ cells | 2.1 | 6.2 | 5.8 | 3.8 | 1.6 | 0.006 | 3.7 | 5.5 |
| 17 | XLA | 37 | 349.5 | <6.7 | copies/μL | 1.7 | 6.6 | 6.0 | 4.6 | 1.2 | 0.009 | 4.5 | 4.9 |
| 18 | XLA | 38 | 45.55 | 0 | copies/μL | 1.6 | 5.2 | 4.9 | 3.9 | 0.9 | 0.02 | 3.2 | 6.7 |
| 19 | XLA | 39 | 2,268 | 0 | copies/10^5^ cells | 1.5 | 4.1 | 4.0 | 3.4 | 0.6 | 0.03 | NA | 4.0 |
| 20 | XLA | 36 | 4,180 | 82 | copies/10^5^ cells | 2.2 | 7.0 | 6.3 | 4.8 | 1.4 | 0.009 | 0.8 | 9.0 |
| 22 | XLA | 38 | 261 | 0 | copies/μL | 1.1 | 5.1 | 4.8 | 3.7 | 0.7 | 0.02 | 4.4 | 7.0 |
| 29 | XLA | NA | 94.3 | 0 | copies/μL | 2.6 | 5.4 | 4.5 | 2.5 | 1.3 | 0.004 | NA | 3.8 |
| 36 | XLA | 38 | 88.3 | 2.8 | copies/μL | 1.4 | 4.3 | 2.1 | 1.8 | 0.3 | 0.01 | NA | 4.5 |
| 37 | XLA | 36 | 42.2 | 3.4 | copies/μL | 0.9 | 3.6 | 3.5 | 2.7 | 0.7 | 0.02 | 2.5 | 2.7 |
| 38 | XLA | 37 | 22.5 | 2.8 | copies/μL | 1.5 | 5.5 | 5.0 | 4.3 | 0.6 | 0.05 | 4.1 | 4.6 |
| 39 | XLA | 39 | 129.5 | 0 | copies/μL | 0.6 | 5.7 | 5.4 | 3.5 | 1.5 | 0.006 | NA | 6.5 |
| 45 | XLA | 41 | NA | NA |  | 4.9 | 6.2 | 4.9 | 3.6 | 0.9 | 0.006 | NA | 8.3 |
| 25 | IKAROS deficiency | 38 | 37.4 | 0 | copies/μL | 2.7 | 4.2 | 3.3 | 2.1 | 1.0 | 0.004 | NA | 2.8 |
| 40 | Agammaglobulinemia | 38 | 6,078 | 0 | copies/10^5^ cells | 3.4 | 7.7 | 6.4 | 4.7 | 1.8 | 0.01 | 3.8 | 8.2 |
| 5 | Heterozygous *FOXN1* | 40 | <6.7 | 144 | copies/μL | 2.4 | 2.6 | 1.1 | 0.6 | 0.6 | 0.6 | NA | 6.9 |
| 7 | Heterozygous *FOXN1* | 37 | 489 | 2,711 | copies/10^5^ cells | 0.9 | 8.1 | 5.4 | 1.2 | 3.6 | 1.7 | 1.1 | 5.9 |
| 8 | Heterozygous *FOXN1* | 37 | 323 | 2,173 | copies/10^5^ cells | 2.5 | 6.4 | 3.9 | 1.2 | 2.4 | 1.9 | NA | 6.5 |
| 12 | Heterozygous *FOXN1* | 38 | 1 | 38 | copies/μL | 3.4 | 0.9 | 0.2 | 0.03 | 0.01 | 0.2 | 0.07 | 7.0 |
| 33 | Heterozygous *FOXN1* | 37 | 3.4 | 24.6 | copies/μL | 1.8 | 1.9 | 1.4 | 1.1 | 0.2 | 0.2 | NA | 9.0 |
| 44 | Heterozygous *FOXN1* | 37 | 0 | 3,265 | copies/10^5^ cells | 3.2 | 1.4 | 0.2 | 0.1 | 0.03 | 0.4 | NA | 9.1 |
| 41 | Heterozygous *FOXN1* | 41 | 0 | 5,653 | copies/10^5^ cells | 3.8 | 1.2 | 0.1 | 0.08 | 0.3 | 0.3 | NA | 7.6 |
| 28 | cDGS | 34 | 0 | 11 | copies/μL | 12.9 | 0.2 | 0.04 | 0.02 | 0.008 | 0.07 | 0.02 | 4.1 |
| 46 | congenital LCH | 37 | 0 | 715 | copies/10^5^ cells | 1.2 | 0.4 | 0.2 | 0.2 | 0.02 | 0.2 | NA | NA |

ADA deficiency, adenosine deaminase deficiency; ALC, absolute lymphocyte count; ANC, absolute neutrophil count; cDGS, complete DiGeorge syndrome; LCH, Langerhans cell histiocytosis; NA, not available; RD, reticular dysgenesis; UPN, unique patient number; X-SCID, X-linked severe combined immunodeficiency; XLA, X-linked agammaglobulinemia
