# Supplementary material for: Nationwide TREC/KREC-based newborn screening and clinical outcomes in Japan
Source: J Hum Immun. 2026 Aug 17;2(5):e20260083. doi: 10.70962/jhi.20260083 (PMC13521547; doi:10.70962/jhi.20260083)
Supplement: Supplementary file 5 [file jhi_20260083_tables5.docx]

**Table S5. Details of hematopoietic cell transplantation (HCT)**

| UPN | Diagnosis | Age at HCT,  days | Graft source | HLA  matching | Conditioning | GVHD prophylaxis | Acute GVHD,  grade | Chronic GVHD | Second HCT, Age at HCT | Engraftment |
| --- | --- | --- | --- | --- | --- | --- | --- | --- | --- | --- |
| 2 | X-SCID | 427 | UR-BMT | 6/8 | BU+FLU | TAC+MTX | III | - | - | Y |
| 3 | X-SCID | 105 | UR-CBT | 7/8 | FLU+MEL | TAC+MTX +MMF | - | - | - | Y |
| 4 | X-SCID | 92 | UR-CBT | 8/8 | BU+FLU | TAC+MTX | - | - | - | Y |
| 6 | X-SCID | 106 | UR-CBT | 8/8 | BU+FLU | TAC+MTX | - | Limited | - | Y |
| 13 | X-SCID | 148 | UR-CBT | 8/8 | BU+FLU | TAC+MTX | - | Limited | - | Y |
| 24 | X-SCID | 104 | UR-CBT | 7/8 | BU+FLU | TAC+MTX | - | - | - | Y |
| 27 | X-SCID | 144 | UR-CBT | 7/8 | BU+FLU | TAC+MTX | - | - | - | Y |
| 32 | X-SCID | 113 | UR-CBT | 8/8 | BU+FLU | TAC+MTX | - | - | - | Y |
| 34 | X-SCID | 119 | UR-BMT | 6/8 | BU+FLU | TAC+MTX | I | - | - | Y |
| 35 | X-SCID | 141 | UR-PBSCT | 8/8 | BU+FLU | TAC+MTX | - | - | - | Y |
| 14 | RD | 173 | UR-CBT | 8/8 | BU+Cy | TAC+MTX | - | Limited | - | Y |
| 42 | RD | 59 | UR-CBT | 8/8 | None | TAC | II | - | 7/8 matched UR-CBT (BU+Cy), day 268 | Y |
| 43 | *JAK3* deficiency | 182 | UR-CBT | 7/8 | None | TAC+MTX | I | - | 7/8 matched UR-CBT (BU+FLU), day 325 | Y |
| 26 | T^-^B^+^NK^+^ SCID | 148 | UR-CBT | 7/8 | BU+FLU | TAC+MTX | III | - | - | Y |

BU, busulfan; Cy, cyclophosphamide; FLU, fludarabine; GVHD, graft-versus-host disease; HLA, human leukocyte antigen; MEL, melphalan; MMF, mycophenolate mofetil; MTX, methotrexate; RD, reticular dysgenesis; TAC, tacrolimus; UPN, unique patient number; UR-BMT, unrelated donor bone marrow transplantation; UR-CBT, unrelated cord blood transplantation; UR-PBSCT, unrelated peripheral blood stem cell transplantation; X-SCID, X-linked severe combined immunodeficiency.
