# Supplementary material for: Nationwide TREC/KREC-based newborn screening and clinical outcomes in Japan
Source: J Hum Immun. 2026 Aug 17;2(5):e20260083. doi: 10.70962/jhi.20260083 (PMC13521547; doi:10.70962/jhi.20260083)
Supplement: Supplementary file 6 [file jhi_20260083_tables6.docx]

**Table S6. Annual coverage of TREC/KREC-based newborn screening in Japan**

| Period | Newborns screened (n) | Population-based  coverage (%) | Regions performing TREC/KREC screening (n) | Regional coverage among NBS-eligible newborns in each region, median (IQR) (%) |
| --- | --- | --- | --- | --- |
| Apr 2020–Mar 2021 | 60,064 | 7.3 | 11 | 20.4 (1.8–45.4) |
| Apr 2021–Mar 2022 | 130,730 | 16.1 | 20 | 61.6 (33.1–72.6) |
| Apr 2022–Mar 2023 | 247,554 | 32.6 | 36 | 54.3 (37.9–73.2) |
| Apr 2023–Mar 2024 | 370,111 | 51.9 | 58 | 74.3 (42.9–82.8) |
| Apr 2024–Mar 2025 | 572,175 | 84.4 | 67 | 86.1 (75.1–97.0) |

IQR, interquartile range; KREC, kappa-deleting recombination excision circle; NBS, newborn screening; TREC, T-cell receptor excision circle.
