# Supplementary material for: Nationwide TREC/KREC-based newborn screening and clinical outcomes in Japan
Source: J Hum Immun. 2026 Aug 17;2(5):e20260083. doi: 10.70962/jhi.20260083 (PMC13521547; doi:10.70962/jhi.20260083)
Supplement: Supplementary file 7 [file jhi_20260083_tables7.docx]

**Table S7. Positive predictive values for TREC/KREC screening assays**

| Assay kits (Manufacturer) | Total births  screened (n) | Number of  Laboratories (n) | Kit-specific PPV, % (95% CI) | |
| --- | --- | --- | --- | --- |
|  |  |  | PPV(SCID/TREC referrals) | PPV(XLA and other  agammaglobulinemias/KREC referrals) |
| NeoMDx (Revvity, USA) | 482,658 | 17 | 2.4 (0.8–5.5) | 5.5 (2.0–11.5) |
| TKSneoFinder (Shimadzu SDC, Japan) | 271,758 | 4 | 3.9 (0.8–11.1) | 2.3 (0.1–12.3) |
| NeoSMAART T/K/S (Sekisui Medical, Japan) | 267,720 | 9 | 1.7 (0.3–4.8) | 4.9 (1.8–10.3) |
| A laboratory-developed assay (KM Biologics Co., Ltd., Japan) | 121,380 | 2 | 0.0 (0.0–14.8) | 6.5 (0.8–21.4) |
| EnLite TREC-KREC (Revvity, USA) | 116,536 | 3 | 6.1 (0.7–20.2) | 0.0 (0.0–11.6) |
| TaqMan SCID/SMA Plus Assay (Thermo Fisher Scientific, USA) | 46,809 | 2 | 16.7 (0.4–64.1) | 0.0 (0.0–13.2) |
| NeoSMAART TREC/KREC (Sekisui Medical, Japan) | 39,688 | 3 | 5.0 (1.2–24.9) | 5.9 (0.1–28.7) |
| TKR (Nihon Techno Service Co., Ltd., Japan) | 34,085 | 1 | 0.0 (0.0–70.8) | 0.0 (0.0–97.5) |

CI, confidence interval; KREC, kappa-deleting recombination excision circle; PPV, positive predictive value; SCID, severe combined immunodeficiency; TREC, T-cell receptor excision circle; XLA, X-linked agammaglobulinemia.
