# Supplementary material for: Nationwide TREC/KREC-based newborn screening and clinical outcomes in Japan
Source: J Hum Immun. 2026 Aug 17;2(5):e20260083. doi: 10.70962/jhi.20260083 (PMC13521547; doi:10.70962/jhi.20260083)
Supplement: Supplementary file 8 [file jhi_20260083_tables8.docx]

**Table S8. Lists of Surveyed Institutions and Newborn Screening Laboratories**

| Category | Institution or laboratory |
| --- | --- |
| Surveyed IEI center | Akita University Hospital |
| Surveyed IEI center | Chiba University Hospital |
| Surveyed IEI center | Dokkyo Medical University Hospital |
| Surveyed IEI center | Ehime University Hospital |
| Surveyed IEI center | Fukuoka University Hospital |
| Surveyed IEI center | Fukushima Medical University Hospital |
| Surveyed IEI center | Gifu University Hospital |
| Surveyed IEI center | Gunma University Hospital |
| Surveyed IEI center | Hamamatsu University Hospital |
| Surveyed IEI center | Hiroshima University Hospital |
| Surveyed IEI center | Hirosaki University Hospital |
| Surveyed IEI center | Hokkaido University Hospital |
| Surveyed IEI center | Hospital of the University of Occupational and Environmental Health |
| Surveyed IEI center | Hyogo Medical University Hospital |
| Surveyed IEI center | Hyogo Prefectural Amagasaki General Medical Center |
| Surveyed IEI center | Hyogo Prefectural Kobe Children's Hospital |
| Surveyed IEI center | Ibaraki Children’s Hospital |
| Surveyed IEI center | Institute of Science Tokyo Hospital |
| Surveyed IEI center | International University of Health and Welfare Hospital |
| Surveyed IEI center | Iwate Medical University Hospital |
| Surveyed IEI center | Jichi Medical University Hospital |
| Surveyed IEI center | Kagawa University Hospital |
| Surveyed IEI center | Kagoshima University Hospital |
| Surveyed IEI center | Kanagawa Children's Medical Center |
| Surveyed IEI center | Kanazawa University Hospital |
| Surveyed IEI center | Kitasato University Hospital |
| Surveyed IEI center | Kobe University Hospital |
| Surveyed IEI center | Kochi University Hospital |
| Surveyed IEI center | Kurume University Hospital |
| Surveyed IEI center | Kyoto Prefectural University of Medicine Hospital |
| Surveyed IEI center | Kyoto University Hospital |
| Surveyed IEI center | Kyushu University Hospital |
| Surveyed IEI center | Mie University Hospital |
| Surveyed IEI center | Nagasaki University Hospital |
| Surveyed IEI center | Nagoya University Hospital |
| Surveyed IEI center | Nara Medical University Hospital |
| Surveyed IEI center | National Center for Child Health and Development |
| Surveyed IEI center | National Defense Medical College Hospital |
| Surveyed IEI center | National Hospital Organization Kumamoto Medical Center |
| Surveyed IEI center | Niigata University Medical & Dental Hospital |
| Surveyed IEI center | Okayama University Hospital |
| Surveyed IEI center | Oita University Hospital |
| Surveyed IEI center | Osaka Metropolitan University Hospital |
| Surveyed IEI center | Osaka University Hospital |
| Surveyed IEI center | Osaka Women's and Children’s Hospital |
| Surveyed IEI center | Saga University Hospital |
| Surveyed IEI center | Saitama Children’s Medical Center |
| Surveyed IEI center | Shiga University of Medical Science Hospital |
| Surveyed IEI center | Shimane University Hospital |
| Surveyed IEI center | Shinshu University Hospital |
| Surveyed IEI center | Shizuoka Children's Hospital |
| Surveyed IEI center | St. Marianna University Hospital |
| Surveyed IEI center | The Jikei University Hospital |
| Surveyed IEI center | Tohoku University Hospital |
| Surveyed IEI center | Tokai University Hospital |
| Surveyed IEI center | Tokushima University Hospital |
| Surveyed IEI center | Tokyo Women’s Medical University Hospital |
| Surveyed IEI center | Tottori University Hospital |
| Surveyed IEI center | Toyama University Hospital |
| Surveyed IEI center | Toyooka Hospital |
| Surveyed IEI center | University of Fukui Hospital |
| Surveyed IEI center | University of Miyazaki Hospital |
| Surveyed IEI center | University of the Ryukyus Hospital |
| Surveyed IEI center | University of Tsukuba Hospital |
| Surveyed IEI center | University of Yamanashi Hospital |
| Surveyed IEI center | Wakayama Medical University Hospital |
| Surveyed IEI center | Yamagata University Hospital |
| Surveyed IEI center | Yamaguchi University Hospital |
| Surveyed IEI center | Yokohama City University Hospital |
| NBS laboratory | Almeida Memorial Hospital |
| NBS laboratory | Aichi Health Promotion Foundation |
| NBS laboratory | Chiba Foundation for Health Promotion and Disease Prevention |
| NBS laboratory | Fukushima Health Preservation Service Association |
| NBS laboratory | Gifu Research Center for Public Health |
| NBS laboratory | Gunma Health Foundation |
| NBS laboratory | Hiroshima City Medical Association Clinical Laboratory |
| NBS laboratory | Hokkaido Pharmaceutical Association Public Health Examination Center |
| NBS laboratory | Hyogo Prefecture Health Promotion Association Public Health Examination Center |
| NBS laboratory | Ibaraki Health Service Association |
| NBS laboratory | Kagawa Health Service Association |
| NBS laboratory | Kagoshima Prefectural Comprehensive Health Center |
| NBS laboratory | Kanagawa Preventive Medicine Association |
| NBS laboratory | KM Biologics Co., Ltd |
| NBS laboratory | Kobe City Medical Association |
| NBS laboratory | Kofu City Medical Association |
| NBS laboratory | Medical Examination Center of Chubu Medical Association |
| NBS laboratory | Miyagi Public Health Association |
| NBS laboratory | Miyazaki Prefectural Health Foundation |
| NBS laboratory | Nagasaki Prefecture Medical Health Association |
| NBS laboratory | Nagano Children’s Hospital |
| NBS laboratory | Niigata Health Service Center |
| NBS laboratory | Okayama Health Foundation |
| NBS laboratory | Osaka City Environmental Health Association |
| NBS laboratory | Osaka Women’s and Children’s Hospital |
| NBS laboratory | Saitama Children’s Medical Center |
| NBS laboratory | Saitama City Institute of Health Science and Public Health |
| NBS laboratory | Shizuoka Preventive Medicine Association |
| NBS laboratory | Shimane University Hospital |
| NBS laboratory | Shimadzu Techno-Research, Inc |
| NBS laboratory | Tochigi Public Health Service Association |
| NBS laboratory | Tokyo Health Service Association |
| NBS laboratory | Toyama Institute of Health |
| NBS laboratory | Yamaguchi Health and Service Association |
| NBS laboratory | Yamanashi Prefectural Central Hospital |

Among the 35 NBS laboratories listed, 32 performed TREC/KREC screening and three provided data from the publicly funded metabolic NBS program only.

IEI, inborn errors of immunity; NBS, newborn screening.
